# The predictive processing embodied in brain conditions: the role of precision

**DOI:** 10.3389/fpsyg.2026.1887747

**Published:** 2026-07-22

**Authors:** A. B. Lao-Rodríguez, S. Cacciato-Salcedo, M. S. Malmierca

**Affiliations:** 1 Cognitive and Auditory Neuroscience Laboratory (CANELAB), Institute of Neuroscience of Castilla y León, University of Salamanca, Salamanca, Spain; 2 Department of Cell Biology and Pathology, University of Salamanca, Salamanca, Spain; 3 Institute for Biomedical Research of Salamanca (IBSAL), Salamanca, Spain

**Keywords:** free-energy principle, mental function and dysfunction, neuromodulatory systems, precision weighting, predictive coding

## Abstract

Predictive coding theory proposes that the brain continuously minimizes prediction errors through hierarchical inference. Increasing evidence, however, suggests that its explanatory power depends not only on prediction generation, but also on precision weighting, the process through which the brain estimates the reliability of sensory, interoceptive, and contextual signals. Within this framework, psychological and psychiatric phenomena may arise from maladaptive precision allocation, leading to altered salience attribution, distorted inference, and inflexible behavioural responses. Neuromodulatory systems are increasingly recognized as critical biological substrates of these processes, regulating the gain assigned to prediction errors across cortical hierarchies. Neurotransmitters such as acetylcholine, dopamine, noradrenaline, and serotonin appear to differentially modulate attention, uncertainty estimation, salience, and contextual updating, thereby shaping perception, learning, and action. From this perspective, psychopathology may be conceptualized as a disorder of embodied precision regulation emerging from disruptions in neuromodulatory control over perceptual, affective, and contextual inference.

## Introduction

**Figure 1:**
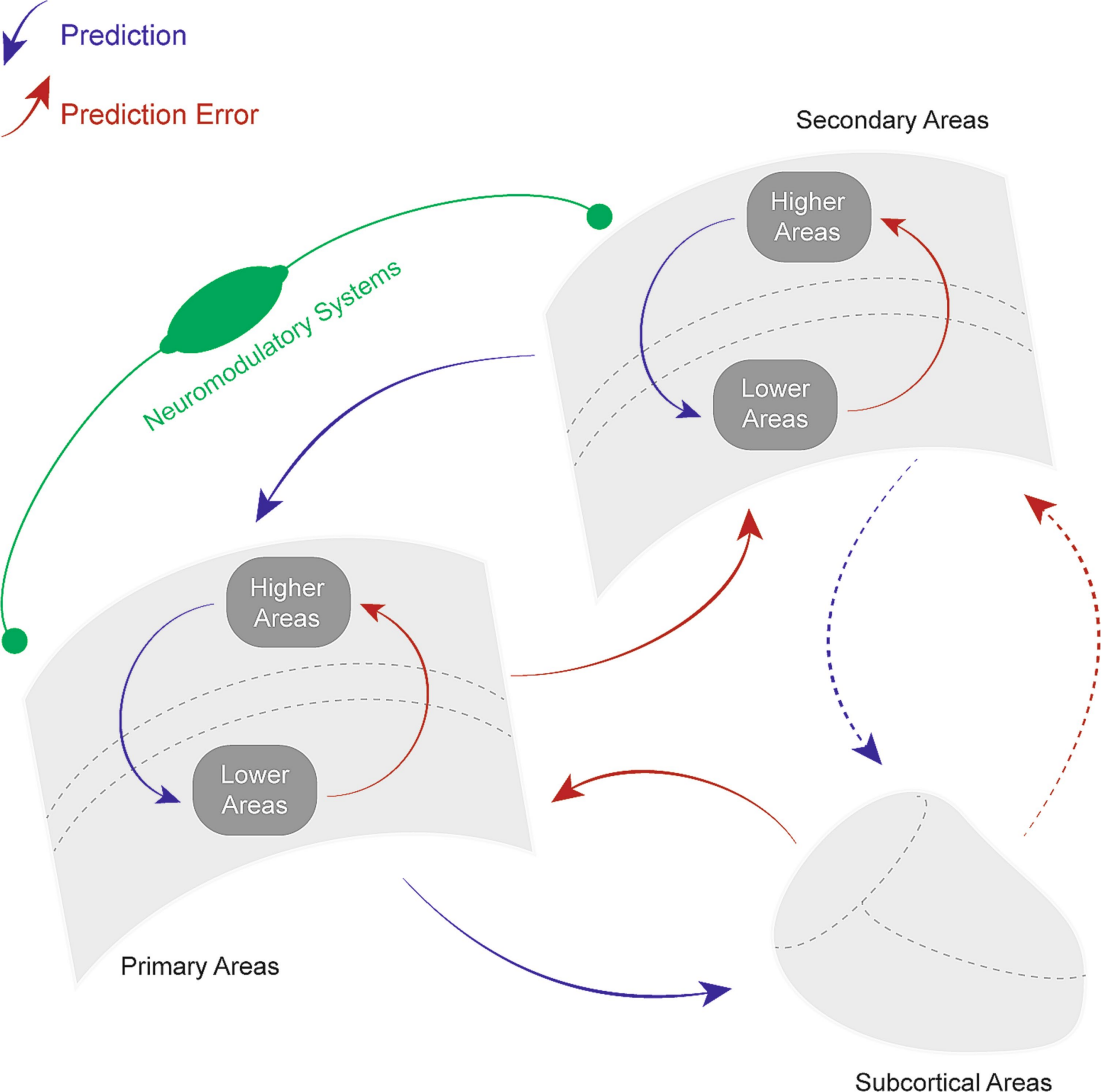
Hierarchical predictive coding scheme. Differences between the expected sensory input and real input generate ascending prediction errors (red arrows) that are transmitted through cortical hierarchies to update higher-level expectations. These expectations generate descending predictions that suppress predictable sensory input (blue arrows). Prediction errors are weighted through the neuromodulatory systems (green) according to their expected precision, such that highly precise signals exert greater influence on perceptual updating, whereas imprecise signals are attenuated. Through this precision-dependent gain control, the system dynamically balances bottom-up sensory evidence and top-down predictions, selectively enhancing informative signals while suppressing unreliable or noisy information. This process contributes to “representational sharpening,” whereby relevant sensory representations become more sensitive to incoming input.

Predictive processing theories propose that the brain does not passively register sensory information but actively anticipates and interprets incoming stimuli through the continuous generation, monitoring, and updating of internal neural models. Two central components of these theories are predictions and prediction errors: predictions correspond to the brain’s expectations about forthcoming sensory input, whereas prediction error signals represent the discrepancies between expected and actual sensory events (Rao and Ballard, 1999; Friston, 2005). Within this framework, higher cortical areas generate top-down predictions that are conveyed to lower hierarchical levels in order to suppress neuronal responses to expected events. When incoming sensory input deviates from current predictions, lower levels transmit bottom-up prediction errors to higher hierarchical levels, enabling the system to update its model of the causes of sensory input (Friston and Kiebel, 2009; Kiebel et al., 2009; Parras et al., 2017; Lao-Rodríguez et al., 2026). Through this recurrent message passing, prediction errors are progressively minimized across the hierarchy (Figure 1).

This framework conceptualizes the brain as a statistical inference machine (Hohwy, 2017; Friston et al., 2021). In contemporary free-energy formulations, this idea is implemented as a biologically plausible account of perceptual inference, in which the brain embodies a generative model of the environment that captures the hierarchical and dynamic structure of sensory input. Neuronal activity is therefore understood as reflecting inference about the hidden causes of sensory events, allowing the brain to predict its own sensations (Mumford, 1992; Rao and Ballard, 1999; Friston, 2010). In Bayesian terms, optimal recognition requires the brain to represent probability distributions over hidden states and environmental causes, updating these representations until they approximate the statistical regularities of the world. Crucially, such inference depends not only on the content of predictions, but also on the precision assigned to prediction errors at different levels of the hierarchy.

This theoretical framework provides compelling accounts of perceptual learning across multiple levels of analysis, including psychology (Knill and Pouget, 2004), cognitive psychophysiology (Winkler et al., 2009; Bendixen et al., 2012), electrophysiology (Rao and Ballard, 1999), and neuroanatomy (Friston, 2005; Antunes and Malmierca, 2011; Lao-Rodríguez et al., 2023). Within this perspective, predictive coding is thought to support not only basic cognitive operations such as perception, but also higher-order functions including short-term memory (Trapp et al., 2021), attention (Feldman and Friston, 2010), hermeneutic and social interpretative processes (Friston and Frith, 2015), and even forms of biological intelligence (Trapp et al., 2025). Consequently, predictive processing has progressively evolved into a unifying theory of brain function, proposing that the brain fundamentally operates by generating hierarchical predictions about sensory input and continuously refining these predictions in light of incoming sensory evidence (Mumford, 1992; Friston, 2005, 2010; Parras et al., 2017).

### Prediction error minimization and precision weighting

Reducing uncertainty through prediction error minimization is a central principle of predictive coding and the free-energy framework. Within this perspective, the brain is understood as an active inferential system that continuously compares incoming sensory information with top-down predictions generated by internal models in order to minimize free energy and maintain adaptive interactions with the environment (Friston, 2005, 2010). When mismatches arise, prediction errors are propagated through hierarchical cortical networks to update beliefs and refine future predictions, allowing perception, learning, and action to collectively reduce uncertainty. When discrepancies between predictions and sensory input cannot be readily resolved, the system engages in information-seeking behaviours—such as orienting movements, attentional shifts, or exploratory actions—to sample additional evidence, refine internal models, and reduce uncertainty (Clark, 2013).

Importantly, this inferential process depends not only on prediction itself but also on the precision assigned to sensory evidence and prediction errors. Precision, often formalized as inverse variance, determines the relative influence of sensory evidence and prior beliefs during perceptual updating. The minimization of prediction error is therefore closely linked to the precision assigned to sensory inputs and internal predictions. Within predictive coding and the free-energy framework, precision acts as a weighting mechanism that determines the reliability or relevance of prediction errors: errors considered more precise exert a stronger influence on the updating of internal models. Thus, the brain not only seeks to reduce discrepancies between expectations and incoming sensory information, but also to estimate how much confidence should be attributed to those discrepancies in order to optimize perception and adaptive responses to the environment (Knill and Pouget, 2004; Feldman and Friston, 2010; Friston, 2010).

The idea that perceptual inference depends on the reliability of available information predates predictive coding formulations and has strong support from psychophysical research. The concept of precision weighting in predictive coding is closely related to earlier Bayesian accounts of perception, which proposed that optimal inference requires sensory information to be weighted according to its uncertainty or reliability (Knill and Pouget, 2004). Within these frameworks, the brain is thought to represent not only estimates of environmental states, but also the uncertainty associated with those estimates, allowing information from different sources to be integrated according to their relative reliability. Consistent with this view, psychophysical studies have demonstrated that human observers combine sensory cues in a near-optimal manner. For example, Ernst and Banks (2002) showed that visual and haptic information are weighted according to their relative reliability, such that more reliable cues exert greater influence on perceptual judgments and the resulting multisensory estimate exhibits lower variance than either modality alone. These findings provide strong empirical support for the notion that perceptual inference depends on uncertainty-weighted integration of sensory evidence. Predictive coding extends this principle by proposing a neurobiological mechanism through which such reliability-weighted inference can be implemented, formalizing reliability as precision and using it to regulate the gain and influence of prediction errors within hierarchical cortical networks.

From this perspective, perception emerges as a dynamic inferential process in which the brain continuously balances top-down predictions with bottom-up sensory signals. Prediction errors carrying high precision are amplified because they are interpreted as more informative about the external world, whereas imprecise or noisy signals are attenuated and given less influence. This mechanism allows the nervous system to efficiently allocate attentional and computational resources toward the most reliable sources of information. In predictive coding models, precision is therefore deeply related to attention, as attentional processes can be understood as the selective enhancement of particular prediction errors that are expected to be behaviourally relevant.

Importantly, the brain does not treat all predictions or prediction errors equally. Instead, both top-down expectations and bottom-up prediction errors are weighted according to their estimated precision or reliability. As Friston proposed, precision controls the relative influence of sensory evidence and prior beliefs by modulating the synaptic gain of neuronal populations encoding prediction errors (Friston, 2010). Thus, prediction errors considered reliable or informative are amplified and propagated through the cortical hierarchy, exerting a stronger influence on perceptual updating and learning, whereas noisy or unreliable errors are attenuated and exert less impact on inference. Conversely, predictions that consistently minimize prediction error become reinforced through synaptic plasticity mechanisms, strengthening the internal generative model and allowing future sensory input to be processed more efficiently. In this sense, the system dynamically assigns greater gain to signals that improve the accuracy of inference while suppressing signals that are inconsistent, noisy, or behaviourally irrelevant (Atiani et al., 2014; Schultz, 2024).

According to the predictive processing framework, not all prediction errors should receive equal treatment because sensory signals may arise from genuine environmental changes, ambiguity, or internal neural noise. Therefore, the brain must continuously discriminate between informative and unreliable prediction errors. Expected precision acts as the mechanism through which reliable signals are amplified while noisy or uncertain signals are attenuated (Spratling, 2008; Feldman and Friston, 2010; Friston, 2012). Only sufficiently precise prediction errors are allowed to substantially update prior beliefs, whereas low-precision signals are down-weighted in favour of stable top-down expectations. Consequently, perception emerges from a dynamic precision-weighted balance between predictions and sensory evidence, enabling the nervous system to optimize inference while minimizing uncertainty and computational cost.

### Neuromodulation as the biological basis of precision

Under the free-energy framework, neuromodulatory systems have been proposed to encode the precision, or certainty, of prediction errors within cortical hierarchies (Friston, 2008; Atiani et al., 2014; Valdés-Baizabal et al., 2020; Pérez-González et al., 2024; Schultz, 2024). Precision is thought to correspond to the synaptic gain of neuronal populations encoding prediction errors, such that highly precise signals are amplified and exert greater influence on perceptual updating and behaviour, whereas low-precision signals are attenuated. In this way, neuromodulation provides a plausible biological mechanism for regulating attention, salience, learning, and adaptive behaviour through gain control of prediction error signaling (Figure 1).

Neuromodulatory systems such as acetylcholine, dopamine, noradrenaline, and serotonin are thought to dynamically regulate the excitability and gain of cortical circuits according to contextual demands and environmental uncertainty (Ayala and Malmierca, 2015; Valdés-Baizabal et al., 2020; Pérez-González et al., 2024; Schultz, 2024; Groves et al., 2025; Mechtenberg, 2026).

By modulating neuronal responsiveness, neuromodulatory systems determine which prediction errors are prioritized and propagated through hierarchical networks, thereby regulating the balance between top-down expectations and bottom-up sensory evidence. Within this framework, neuromodulation is thought to encode the expected reliability of perceptual inferences rather than the content of perception itself, effectively signaling how much confidence should be assigned to sensory representations. This perspective has led predictive processing theories to conceptualize attention as a higher-order process of precision weighting, whereby attended signals are those expected to be most informative and behaviourally relevant (Feldman and Friston, 2010; Parr and Friston, 2019).

Among neuromodulatory systems, acetylcholine has been strongly associated with attentional modulation, sensory uncertainty, and precision weighting (Yu and Dayan, 2002; Herrero et al., 2008; Ayala and Malmierca, 2015; Pérez-González et al., 2024; Tu et al., 2025). Experimental evidence from the auditory cortex demonstrates that cholinergic modulation selectively enhances neuronal mismatch responses to deviant auditory stimuli while sparing repetition suppression mechanisms. This selective amplification of prediction error responses has been interpreted as consistent with increased precision weighting of unexpected sensory signals and enhanced access of informative prediction errors to higher cortical levels. Such findings suggest that acetylcholine may play a central role in gating the propagation of sensory prediction errors across hierarchical auditory networks.

Dopamine has also been extensively linked to precision encoding, particularly in relation to motivational salience, reward prediction, and action selection (Friston, 2012; Lak et al., 2016; Schultz, 2024). Within active inference formulations, dopamine has been proposed to encode the expected precision of policies and action-related prediction errors, thereby regulating confidence in behavioural strategies and goal-directed action. Alterations in dopaminergic precision signaling may therefore profoundly influence belief updating, salience attribution, and reinforcement learning.

Noradrenergic systems are thought to contribute to the estimation of environmental volatility and contextual uncertainty, dynamically regulating arousal and attentional flexibility under changing conditions (Aston-Jones and Cohen, 2005; Mechtenberg, 2026). This proposal is closely aligned with Bayesian models of learning in which estimates of environmental volatility determine the rate at which beliefs are updated. Behrens et al. (2007) demonstrated that human learners dynamically adapt their learning rates according to the perceived stability of the environment, increasing the influence of new information when contingencies become volatile and reducing it when environmental statistics remain stable. Building upon this framework, the Hierarchical Gaussian Filter (HGF) formalized learning as a hierarchical process in which belief updating is driven by precision-weighted prediction errors and higher-order estimates of uncertainty and volatility (Mathys et al., 2014). Within this model, volatility acts as a latent variable governing environmental uncertainty and dynamically modulating the impact of prediction errors on belief updating. Importantly, these computational approaches provide quantitative estimates of uncertainty, volatility, and precision, offering a formal operationalization of concepts that are often difficult to measure directly. From this perspective, noradrenergic signaling may contribute to the dynamic adjustment of learning rates by encoding changes in environmental uncertainty and influencing the precision assigned to prediction errors. Similarly, serotonin has increasingly been implicated in affective inference, interoceptive processing, and the regulation of uncertainty associated with emotional and bodily states. Together, these neuromodulatory systems appear to shape the precision landscape through which the brain determines which signals should dominate perception, learning, and action.

Crucially, these precision-weighting processes are deeply embodied. Interoceptive states, autonomic signals, and bodily sensations continuously interact with environmental and social context to shape how uncertainty is interpreted and acted upon. From this perspective, neuromodulatory systems may provide a mechanistic bridge between body, brain, and environment by dynamically regulating the salience and reliability assigned to sensory, affective, and interoceptive signals.

### Maladaptive precision weighting in clinical conditions

Recent evidence has increasingly extended predictive coding accounts beyond basic sensory processing, showing that alterations in precision weighting and hierarchical inference may underlie neurodevelopmental and psychiatric conditions such as autism and schizophrenia.

In schizophrenia research, aberrant precision weighting has been proposed as a key mechanism underlying maladaptive perceptual inference and impaired reality monitoring. According to Raza and colleagues, impairments in top-down and bottom-up integration may produce unstable auditory representations, causing individuals either to fail to register salient deviations or over-respond to irrelevant variability (Raza et al., 2025). The authors further connect these abnormalities to disrupted mismatch negativity (MMN) responses and to the NMDA hypofunction hypothesis, highlighting evidence that reduced NMDA receptor activity weakens prediction-error signaling and alters the brain’s ability to update internal models (Raza et al., 2025). This perspective aligns with earlier predictive processing accounts suggesting that psychotic symptoms may arise when abnormal precision is assigned either to sensory prediction errors or to higher-order priors, thereby distorting the interpretation of sensory experience (Fletcher and Frith, 2009; Adams et al., 2013; Katthagen et al., 2022).

Similar computational principles have also been applied to autism spectrum disorder. Arthur et al. reviewed competing predictive coding theories of autism and argued that autistic perception may not be best explained by globally weakened priors, but rather by atypical encoding of precision and impaired context-sensitive updating of learning rates (Arthur et al., 2023). Their active inference modeling showed that autistic individuals did not demonstrate generalized deficits in prediction itself but instead showed difficulties flexibly adapting learning rates in response to environmental volatility. The authors therefore proposed that autistic perception may involve altered precision assignment during uncertain or dynamically changing contexts rather than a simple attenuation of priors (Arthur et al., 2023). This interpretation is further supported by studies at the neuronal level (Cacciato-Salcedo et al., 2026).

Extending this line of work, Adamou et al. developed a multi-scale mathematical framework integrating predictive coding, information theory, and network neuroscience to explain the dynamic nature of autism symptoms (Adamou et al., 2026). Their framework proposes that excessive precision assigned to low-level sensory prediction errors may contribute to heightened perceptions of unpredictability and environmental entropy. Within this model, behaviours such as insistence on sameness, repetitive actions, and sensory sensitivities are interpreted not merely as deficits, but as adaptive strategies for reducing uncertainty and minimizing overwhelming prediction error signals. The authors additionally connect these mechanisms to atypical long-range neural connectivity and increased cognitive load during social interaction, proposing that social fatigue and sensory overload emerge from the continuous requirement to resolve imprecise or unstable predictions (Adamou et al., 2026).

Contemporary predictive coding accounts increasingly suggest that psychological and psychiatric phenomena cannot be fully understood solely in terms of inaccurate predictions, but rather through dynamic alterations in the balance of precision assigned to bodily, perceptual, and contextual signals. Within this framework, the brain continuously estimates not only what is likely to occur, but also which sources of information should be trusted at any given moment. Precision weighting therefore operates as a regulatory mechanism that shapes attention, salience, emotional experience, and behavioural adaptation by modulating the relative influence of prior beliefs and incoming sensory evidence.

An important challenge for precision-based accounts concerns the extent to which alterations in precision can be identified independently of the behavioural phenomena they are intended to explain. If precision is inferred retrospectively from clinical symptoms alone, predictive coding risks becoming overly flexible and difficult to falsify (Bowman et al., 2023). Recent advances in computational psychiatry have begun to address this limitation by providing formal methods for estimating latent variables such as uncertainty, volatility, learning rates, and precision. Hierarchical Bayesian frameworks, including the Hierarchical Gaussian Filter (Mathys et al., 2014), allow precision-related processes to be quantified through computational parameters derived from behavioural data. Importantly, converging evidence from neurophysiology suggests that these computational constructs may also be linked to independent biological measures. For example, mismatch negativity (MMN) has been interpreted as a neural marker of prediction-error signaling within hierarchical predictive coding architectures (Fong et al., 2020; Kirihara et al., 2020), while Dynamic Causal Modelling studies propose that intrinsic connectivity parameters may reflect the precision assigned to prediction errors relative to prior beliefs (Kirihara et al., 2020). More recently, pupillometry studies have shown that pupil responses track information gain and precision-weighted prediction errors during belief updating (Colizoli et al., 2026). Although no single biomarker provides a direct measure of precision, these approaches offer increasingly constrained and testable ways of linking predictive coding models to observable neurobiological processes.

From this perspective, psychopathology may emerge when precision allocation becomes maladaptive or inflexible, leading to persistent biases in the interpretation of internal or external signals. Anxiety disorders, for example, may involve hyperprecision assigned to threat-related bodily cues, whereas psychosis may reflect aberrant precision attributed to internally generated predictions. Mental disorders can therefore be conceptualized not simply as cognitive dysfunctions, but as disturbances in the dynamic regulation of precision across body, perception, and context.

## Concluding remarks

Although predictive coding provides a mathematically coherent framework for understanding how biological systems minimize free energy, its translation to the biological brain remains challenging, particularly at synaptic, circuit, and systems levels. Important conceptual and empirical questions persist regarding how predictive computations are implemented neurobiologically, how precision weighting is encoded within neural hierarchies, and how neuromodulatory systems regulate prediction error signaling. One major challenge concerns the operationalization of precision itself. While predictive coding formally describes precision as inverse variance and associates it with synaptic gain, its direct neurobiological correlates remain difficult to identify. Current studies often infer precision indirectly through electrophysiological markers, oscillatory activity, behavioural variability, or neuromodulatory dynamics, but reliable biomarkers are still lacking.

Additional challenges arise from the heterogeneity of predictive coding formulations. Different models vary substantially in their assumptions regarding hierarchical organization, neural circuitry, and computational mechanisms, while the physiological plausibility of canonical predictive architectures remains debated (Milkowski and Litwin, 2022). Similarly, although neuromodulatory systems are frequently proposed to implement precision weighting, the specific contributions and interactions of different neurotransmitters remain incompletely understood. From a clinical perspective, linking computational mechanisms such as aberrant precision weighting to specific psychiatric diagnoses also remains complex, given the heterogeneity and overlap of perceptual, affective, interoceptive, and cognitive symptoms across disorders.

Despite these limitations, predictive coding continues to provide a powerful integrative framework linking perception, action, emotion, neuromodulation, and psychopathology within a common inferential architecture. Increasing evidence suggests that the brain operates not only by minimizing prediction error, but by dynamically regulating the precision assigned to predictions and sensory evidence. Neuromodulatory systems appear to constitute a key biological substrate of this process by regulating synaptic gain and controlling the propagation of prediction errors across cortical hierarchies. From this perspective, psychopathology may reflect disturbances in the dynamic regulation of precision across bodily, sensory, emotional, and contextual domains. Understanding how precision weighting emerges from the interaction between predictive inference, neuromodulation, and embodiment may therefore provide a promising path toward a more mechanistic and integrative account of mental function and dysfunction.
